# Emergence of epidemic O'nyong-nyong fever in southwestern Uganda, after an absence of 35 years.

**DOI:** 10.3201/eid0301.970112

**Published:** 1997

**Authors:** E B Rwaguma, J J Lutwama, S D Sempala, N Kiwanuka, J Kamugisha, S Okware, G Bagambisa, R Lanciotti, J T Roehrig, D J Gubler


### Emergence of Epidemic O'nyong-nyong Fever in Southwestern Uganda, After an Absence of 35 Years

**To the Editor:** In July 1996, an uncommon disease suspected to be O'nyong-nyong fever was recognized in the Rakai district of southwestern Uganda. It was reported to have started in June 1996. The disease spread into the neighboring Mbarara and Masaka districts of Uganda and in the bordering Bukoba district of northern Tanzania.

The initial symptoms of O'nyong-nyong fever are high fever and generalized maculopapular skin rash with crippling arthritis, primarily in the big joints, in the absence of joint effusion. Other features are lymphadenitis, eye pain and reddening with no discharge, chest pain, and general malaise. The disease is self-limiting. All age groups and both sexes are equally affected. In areas where the disease is epidemic, 60% to 80% of the people are infected, and familial clustering is found in affected households. No deaths have been reported, but two miscarriages have been associated with infection.

The Ministry of Health (Uganda), in collaboration with the Uganda Virus Research Institute, began epidemiologic and clinical investigations of the epidemic in August 1996. Acute-phase serum samples were collected from patients, and adult mosquitoes were collected from within and around patients' homes. Virus isolates were made from acute-phase serum samples from several patients by intracranial inoculation and passage in baby mice. Attempted virus isolations from mosquito specimens are in progress. Serum samples and aliquots of the virus isolates were sent to the Centers for Disease Control and Prevention, Fort Collins, Colorado, USA, for reisolation and identification. A portion of the capside and NS4 genes of the virus isolates was sequenced and identified as O'nyong-nyong virus; the virus was isolated and sequenced directly from another serum sample. Two serum samples were positive for IgM antibody to O'nyong-nyong antigen.

O'nyong-nyong virus was responsible for a similar epidemic in 1959 to 1961, which started in northern Uganda and spread south and eastward into Kenya, Tanzania, and Zambia, and then northward from Tanzania into southwestern Uganda, where it subsided. The disease has reemerged in this area after 35 years of absence.

**E.B. Rwaguma,* J.J. Lutwama,* S.D.K. Sempala,* N. Kiwanuka,† J. Kamugisha,‡ S. Okware,‡ G. Bagambisa,§ R. Lanciotti,¶ J. T. Roehrig,¶ and D.J. Gubler¶**

*Uganda Virus Research Institute, Entebbe, Uganda; †Rakai Project Uganda Virus Research Institute; ‡Communicable Disease Control, Ministry of Health, Uganda; §Ministry of Health, Rakai, Uganda; ¶Centers for Disease Control and Prevention, Fort Collins, Colorado, USA

### Prostatitis and Benign Prostatic Hyperplasia: Emerging Infectious Diseases?

**To the Editor:** In their excellent article, Molecular Approaches to the Identification of Unculturable Infectious Agents, Gao and Moore (1) point out that molecular approaches should be unleashed on diseases such as sarcoidosis, Kawasaki disease, and type I diabetes mellitus, which are thought but not proven to be infectious. The authors, however, are overlooking the more common and most likely infectious disease of unknown etiology today—prostatitis.

According to the pathologist McNeal, the prostate gland is the most commonly diseased internal organ of the human body (2). Prostatitis is the most common prostate disease, resulting in more physician visits than either benign prostatic hyperplasia or prostate cancer, according to the National Institutes of Health (3). Despite its frequency, prostatitis as a disease and as a histologic lesion is understudied (4).

By the Meares and Stamey culture localization procedure, in which the first voided urine, a midstream urine, the expressed prostatic secretions, and a final voided urine are compared, more than 90% of cases in patients with chronic pelvic symptoms are labeled as "nonbacterial" prostatitis or prostatodynia, both of which are thought to be incurable diseases (5).

The University of Washington has documented white blood cell counts as high as 38,000 per $mm^3$, in "nonbacterial" prostatitis patients (6). According to urologist Thomas Stamey, up to 50% of all men experience symptoms of prostatitis during

